# Supplementary material for: Low-field onset of Wannier-Stark localization in a polycrystalline hybrid organic inorganic perovskite
Source: Nat Commun. 2021 Sep 29;12:5719. doi: 10.1038/s41467-021-26021-4 (PMC8481244; doi:10.1038/s41467-021-26021-4)
Supplement: Supplementary file 1 — Supplementary Information [file 41467_2021_26021_MOESM1_ESM.pdf]

Supplementary Information

Low-field Onset of Wannier-Stark Localization in a Polycrystalline Hybrid Organic Inorganic Perovskite

Daniel Berghoff[$1], Johannes Bühler[$2], Mischa Bonn[3], Alfred Leitenstorfer[2], Torsten Meier[*1], Heejae Kim[*3]

[1] Department of Physics, Paderborn University, D-33098 Paderborn, Germany

[2]Department of Physics and Center for Applied Photonics, University of Konstanz, D-78457 Konstanz, Germany

[3]Department of Molecular Spectroscopy, Max Planck Institute for Polymer Research, D-55128 Mainz, Germany

[$] equal contribution

Supplementary Note 1

The detailed processes of synthesizing polycrystalline film of $MAPbI_3$ perovskite have been fully described in these literatures, Ref[1,2].

Supplementary Method

In order to investigate the influence of intense THz fields, we take the possible virtual and real carriers generated by multi-photon transitions into account. For that purpose, we solve the complete semiconductor Bloch equations (SBE) for two bands, where transitions originating from the strong THz pulse are taken into account to arbitrary order and the weak optical pulse enters into the equations only in first order, Ref [3].

$$\frac{\partial}{\partial t}p_k^{cv} = \frac{i}{\hbar}(\varepsilon_k^c - \varepsilon_k^v)p_k^{cv} + \frac{e}{\hbar}E_{\mathrm{THz}}(t)\nabla_k p_k^{cv} - \frac{i}{\hbar}E_{\mathrm{THz}}(t)\mu_k^{vc}(1-2n_k^c) - \frac{p_k^{cv}}{T_2} \quad (1)$$

$$\frac{\partial}{\partial t}n_k^c = \frac{e}{\hbar}E_{\mathrm{THz}}(t)\nabla_k n_k^c + \frac{i}{\hbar}E_{\mathrm{THz}}(t)\mu_k^{vc}(p_k^{cv} - (p_k^{cv})^*) - \frac{n_k^c}{T_1} \quad (2)$$

$$\frac{\partial}{\partial t}\delta p_k^{cv} = \frac{i}{\hbar}(\varepsilon_k^c - \varepsilon_k^v)\delta p_k^{cv} + \frac{e}{\hbar}E_{\mathrm{THz}}(t)\nabla_k \delta p_k^{cv} + \frac{e}{\hbar}E_{\mathrm{opt}}(t)\nabla_k p_k^{cv}$$
$$-\frac{i}{\hbar}E_{\mathrm{opt}}(t)\mu_k^{vc}(1-2n_k^c) + \frac{i}{\hbar}E_{\mathrm{THz}}(t)\mu_k^{vc}2\delta n_k^c - \frac{\delta p_k^{cv}}{T_2} \quad (3)$$

$$\frac{\partial}{\partial t}\delta n_k^c = \frac{e}{\hbar}E_{\mathrm{THz}}(t)\nabla_k \delta n_k^c + \frac{e}{\hbar}E_{\mathrm{opt}}(t)\nabla_k n_k^c + \frac{i}{\hbar}E_{\mathrm{THz}}(t)\mu_k^{vc}(p_k^{cv} - (p_k^{cv})^*)$$
$$+\frac{i}{\hbar}E_{\mathrm{opt}}(t)\mu_k^{vc}(\delta p_k^{cv} - (\delta p_k^{cv})^*) - \frac{\delta n_k^c}{T_1} \quad (4)$$

As discussed in the main text, the optical Stark contribution and the intraband acceleration contribution (which leads to Franz Keldysh effect and further Wannier Stark localization in strong fields) can be separated by solving the equation with and without all the intraband THz terms of the kind, $\frac{e}{\hbar}E_{\mathrm{THz}}(t)\nabla_k$, i.e., without these terms we can isolate the optical Stark effect. The strength of the transition dipole moment μ_k^{vc} is determined as described below (Supplementary Fig. 6(a)), and the relaxation time T_1 is chosen to be 20 fs as the dephasing time T_2.

We compare the results from the full SBE (described above) with that from the simple *p*-equation (Eq. (1) in the Method section) in Supplementary Fig. 6 (b). The isolated contributions from the optical Stark effect are presented in Supplementary Fig. 6 (c) and they are compared with the results which also include the Wannier-Stark localization in Fig. 6 (d). This comparison clearly demonstrates that magnitude of the optical Stark shift is below 10meV for the considered fields, whereas the shifts and further modifications of the absorption spectra due to Wannier-Stark localization strongly dominate the response.

To determine the strength of the transition dipole moment μ_k^{vc}, we compare two models as follows.

Model (i), the *p*-equation in the SBE for a three-dimensional k-space including a contact Coulomb potential of strength V_0 that approximates the Coulomb interaction, Ref [4,5]:

$$\frac{\partial}{\partial t}p_{\mathbf{k}}^{cv} = \frac{i}{\hbar}(\varepsilon_{\mathbf{k}}^c - \varepsilon_{\mathbf{k}}^v)p_{\mathbf{k}}^{cv} - \frac{i}{\hbar}E_{\mathrm{opt}}(t)\mu_{\mathbf{k}}^{vc} - \frac{p_{\mathbf{k}}^{cv}}{T_2} + \frac{i}{\hbar}V_0\sum_{\mathbf{k}} p_{\mathbf{k}}^{cv} \quad (5)$$

with $\varepsilon_{\mathbf{k}}^c - \varepsilon_{\mathbf{k}}^v = \varepsilon_{\mathrm{gap}} + \frac{\hbar^2}{2m^*}k^2$ and $m^* = 0.11m_0$ (average value from Ref[6]).

V_0 is chosen in a way, that the 1s exciton peak has a binding energy of 11 meV. The interband transition dipole moment $\mu_{\mathbf{k}}^{vc}$ is taken to be constant in k-space and determines the strength of the 1s exciton peak (quadratic dependence, see e.g. Elliot formula, Ref [7]).

Model (ii), the p-equation for a TLS to model the exciton transition:

$$\frac{\partial}{\partial t} p_{\text{exc.}} = \frac{i}{\hbar}(\varepsilon_{\text{gap}} - \Delta\varepsilon) p_{\text{exc.}} - \frac{i}{\hbar} E_{\text{opt}}(t) \mu_{\text{exc.}} - \frac{p_{\text{exc.}}}{T_2} \tag{6}$$

with $\Delta\varepsilon = 11$ meV and $\mu_{\text{exc.}} = 46$ D.

Here, we use the transition dipole moment between the ground and a non-degenerate exciton state to be $\mu_{\text{exc.}} = 46$ D$(\approx 9.58$ eÅ$)$, the band gap $\varepsilon_{\text{gap}} = 1.642$ eV, and the exciton binding energy about 11 meV, taken from the experimental data from the literature, Ref [8].

Both models are probed with a short optical pulse and the absorption spectra are computed. The comparison of the peak amplitudes in the absorption spectra gives an estimate of the dipole transition moment (Supplementary Fig. 6(a)) for which we estimate a value to $\mu_{\mathbf{k}}^{vc} \approx 0.92$ eÅ.

Supplementary Figures

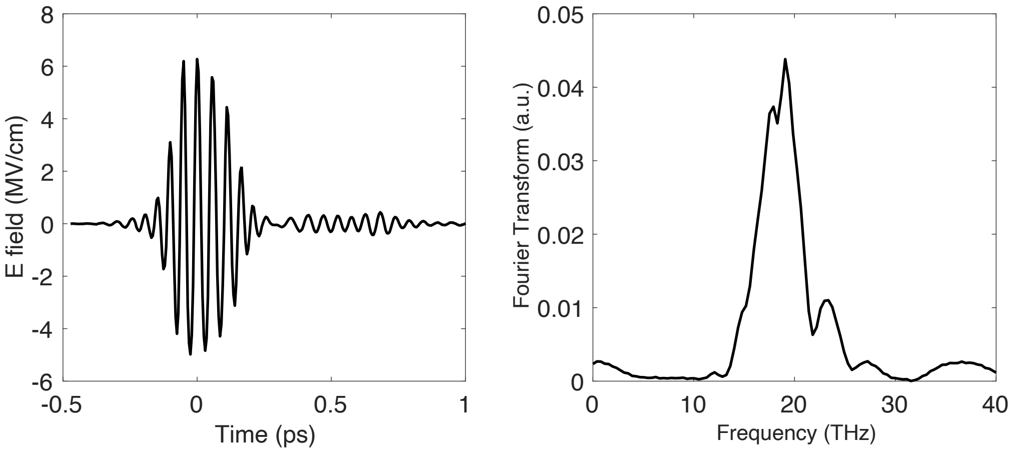

Supplementary Figure 1 Temporal profile of the applied THz bias transient (left) and the Fourier transform of the profile (right) for obtaining the Figure 2 and 4 (b). The full width at half maximum is about 4 THz.

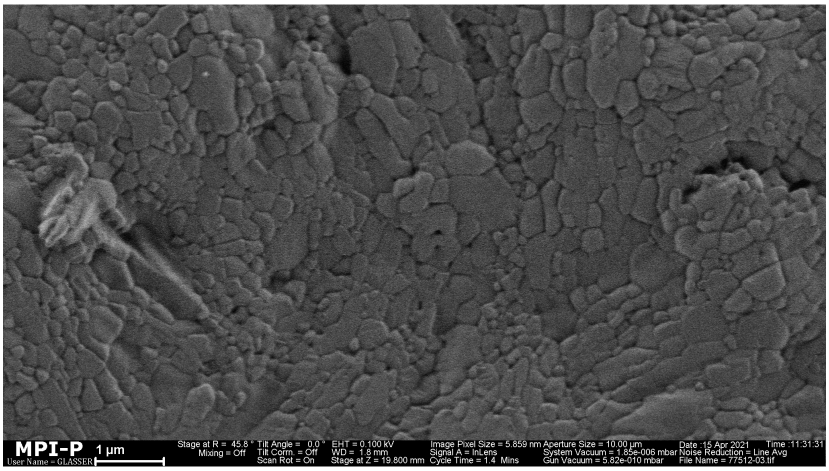

Supplementary Figure 2 Representative SEM images of $MAPbI_3$ perovskite film on TOPAS, 1 µm scale showing micron sized crystallites.

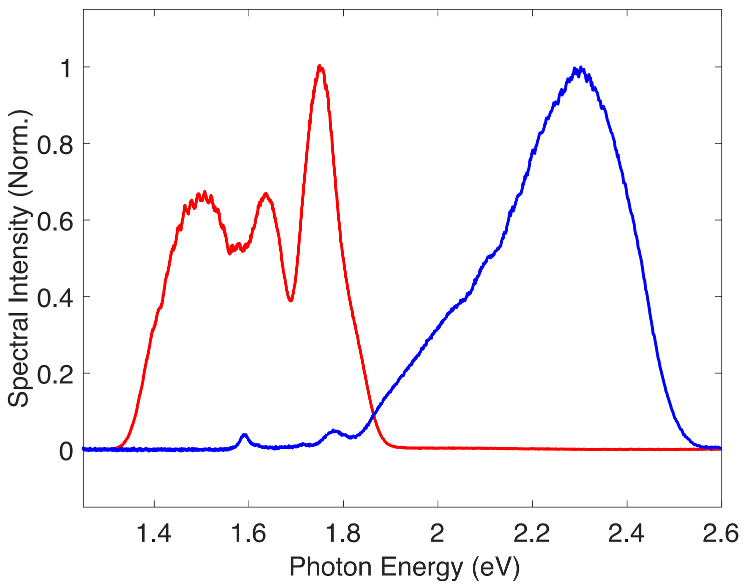

Supplementary Figure 3 Normalized spectra of near-IR (red) and visible (blue) probe pulses generated via non-collinear optical parametric amplification.

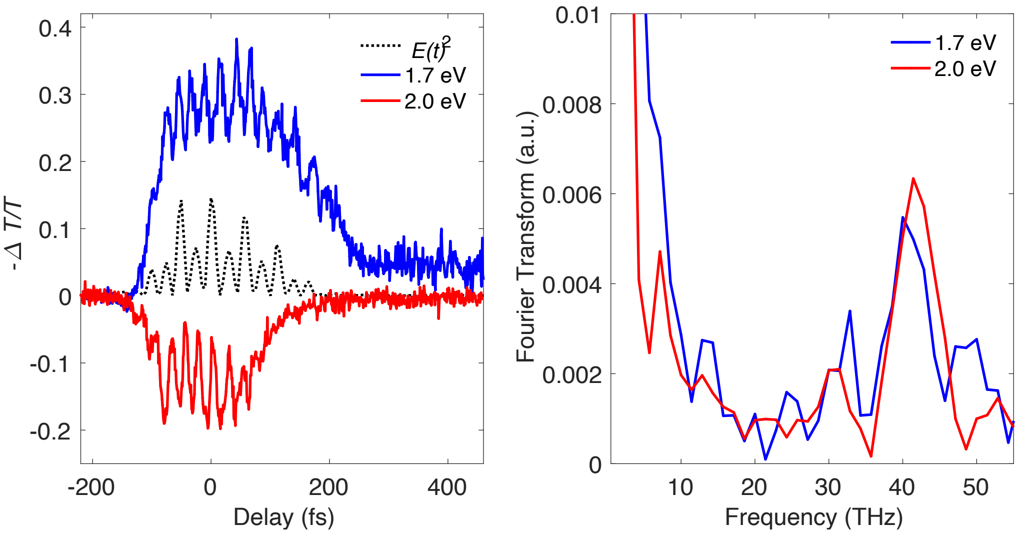

Supplementary Figure 4 Differential transmission changes upon applying a 6 MV/cm THz field measured at probe photon energies of 1.7 eV (blue line) and 2.0 eV (red) together with the $E^2(t)$ of THz pulse profile (left). The Fourier transform of the time trace of the differential transmission changes upon applying a 6 MV/cm THz field measured at probe photon energies of 1.7 eV and 2.0 eV (right).

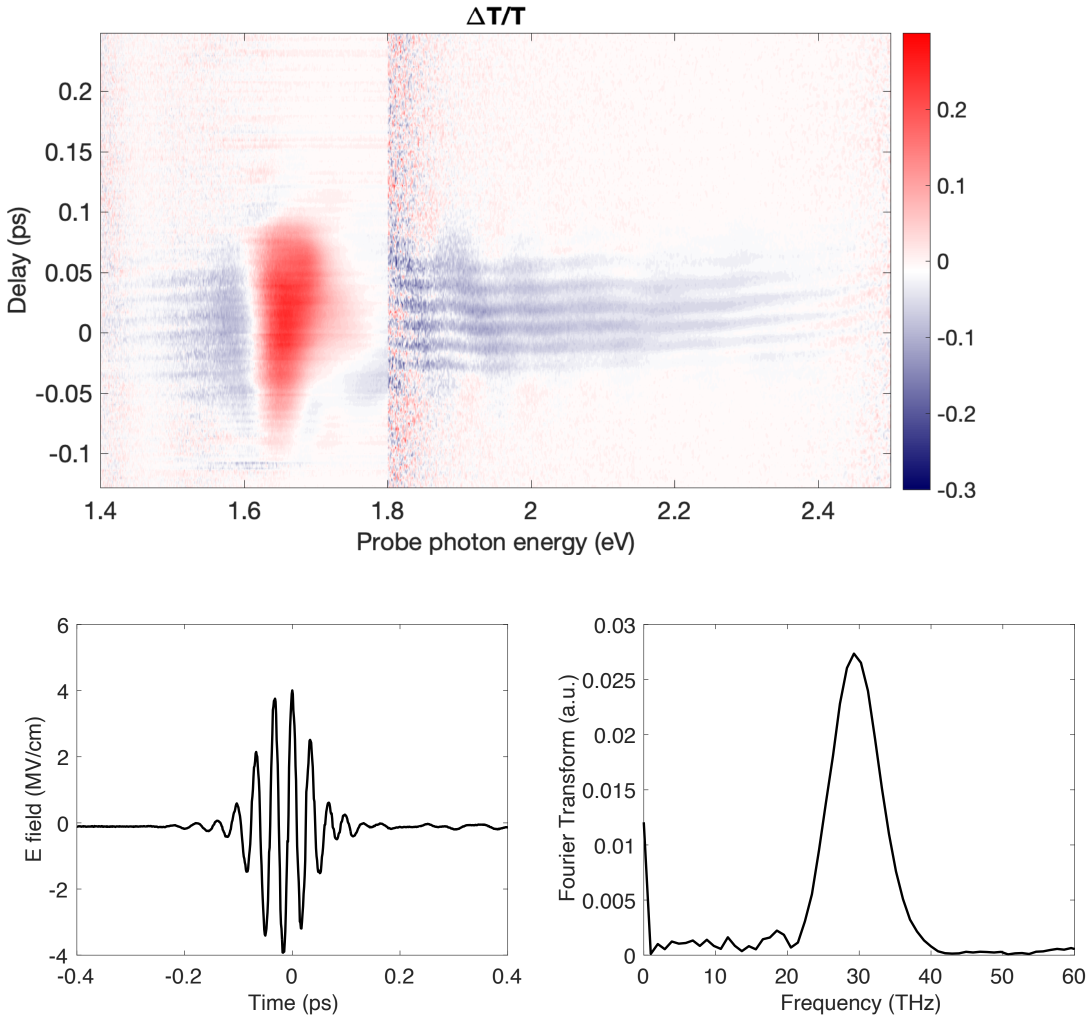

Supplementary Figure 5 Experimental differential transmission spectra on a polycrystalline film of $MAPbI_3$ perovskite at room temperature, as a function of delay time of probe pulses after THz pump pulses (upper). The THz pulses have a peak field strength of 4 MV/cm and a center frequency of 30 THz (lower). The probe pulses have photon energy of 1.4 ~ 2.4 eV.

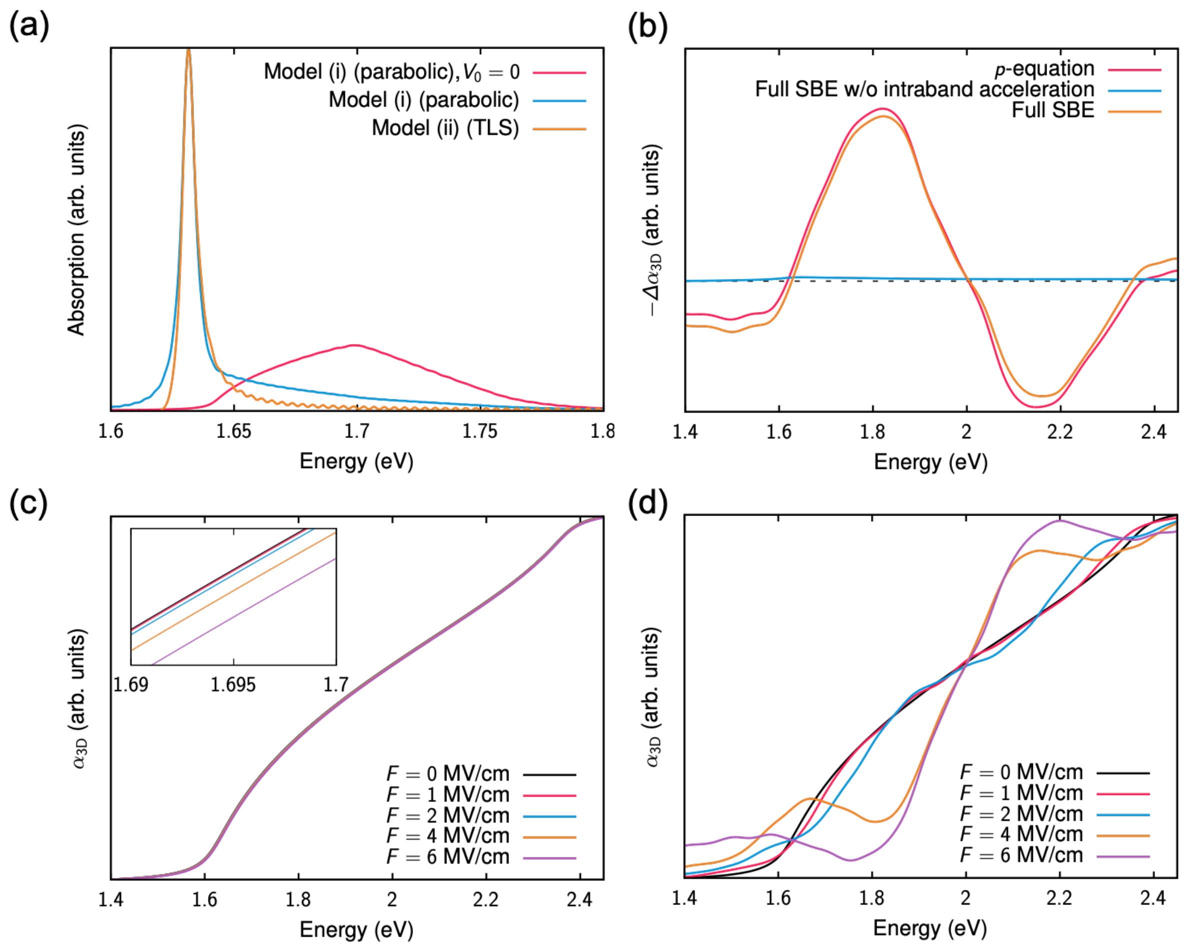

Supplementary Figure 6 (a) Comparison of optical absorption spectra from model (i) and model (ii), see Supplementary Method for details. The peak amplitudes match for a transition dipole moment of $\mu_{\mathbf{k}}^{vc} \approx 0.92$ eÅ. (b) Negative differential absorption spectra for the different models with $\mu_{\mathbf{k}}^{vc} = 1$ eÅ and a peak electric field of 6 MV/cm. The spectra are dominated by the WS effect. There is hardly any difference between the simple p-equation (pink curve) and the full model which takes interband excitations by the THz field non-perturbatively into account (orange curve). When the intraband acceleration, i.e., Wannier-Stark localization, is neglected the resulting absorption changes which originate from the optical Stark effect are very small (blue curve). (c) Absorption spectra for full SBE without any intraband acceleration terms and different field strengths. The inlet shows a small energy range of 10 meV to reveal the magnitude of the optical Stark shift, which is only a couple of meV. (d) Absorption spectra for full SBE at different field strengths.

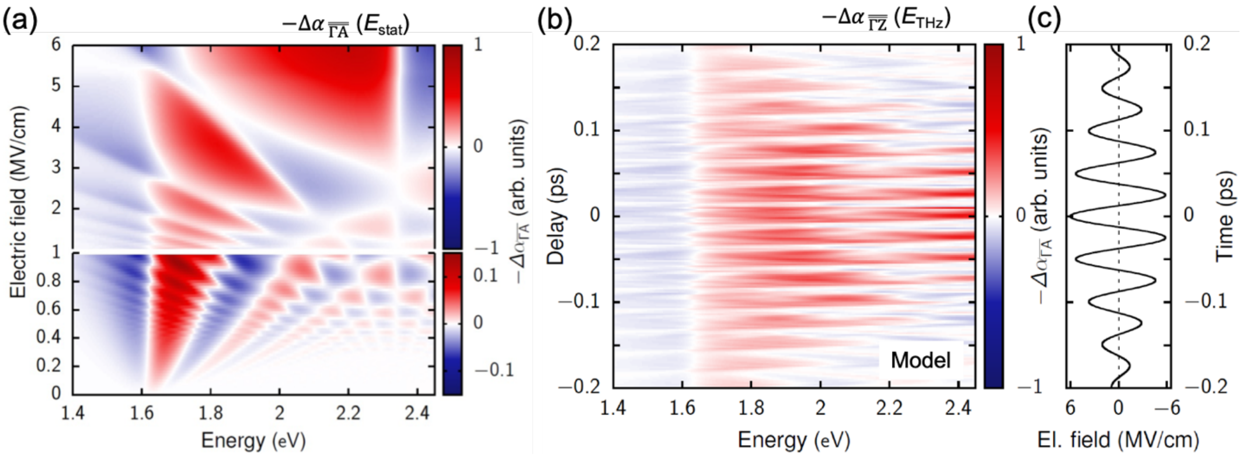

Supplementary Figure 7 (a) Negative change of the optical interband absorption $-\Delta\alpha_{\overline{\Gamma A}}$ for static fields from a cosine band modeling along $\overline{\Gamma A}$ direction. The region of electric field strengths up to 1 MV/cm is enlarged to show Franz-Keldysh oscillations and the transition to the Wannier-Stark regime. (b) Calculated $-\Delta\alpha_{\overline{\Gamma A}}$ spectra for the excitation with a THz pulse with a peak field strength of E_0 = 6 MV/cm, see (c), where the delay τ between the THz and the optical pulse is varied. The pulse duration $\bar{T}$ is 240 fs, the THz frequency is 20 THz, and the dephasing time is T_2 = 20 fs. In both figures S6(a) and S6(b) the respective normalizations of figures 3(a) and 3(b) were used for better comparability which shows that the absorption changes arising from the $\overline{\Gamma A}$ direction are weaker than for the $\overline{\Gamma Z}$ direction, due to the spread over a broader bandwidth.

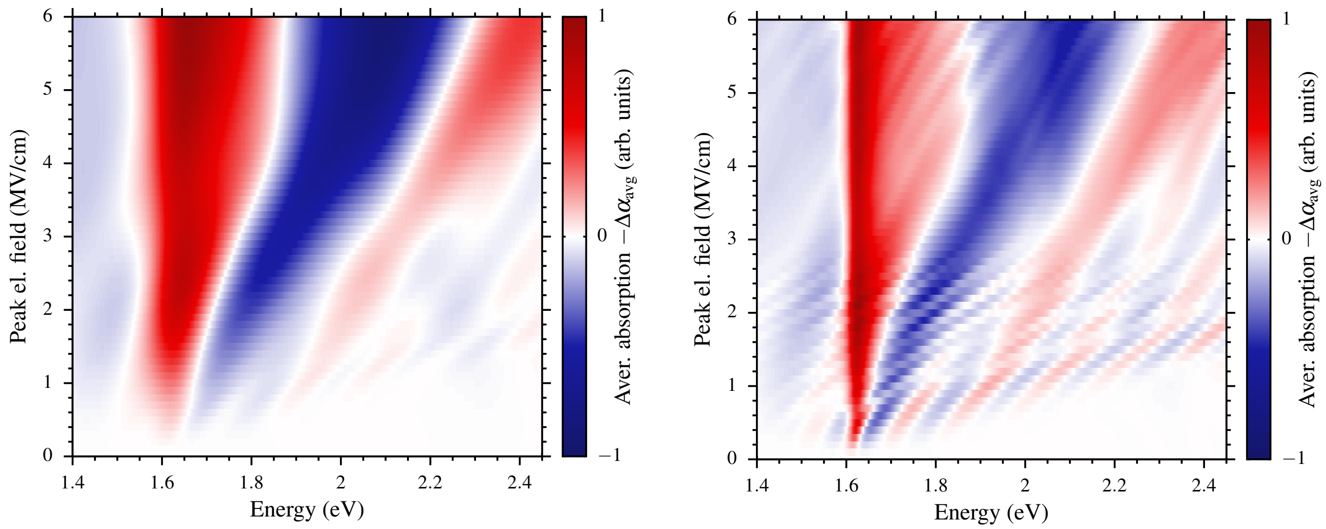

Supplementary Figure 8 Simulations with averaging from the $\overline{\Gamma Z}$ to the $\overline{\Gamma A}$ direction for a THz pulse centered at $t = 0$ and various field strengths. **(a)** averaged 1D absorption change, $-\Delta\alpha_{\text{avg}}$, for $T_2 = 10$ fs. **(b)** averaged 1D absorption change, $-\Delta\alpha_{\text{avg}}$, for $T_2 = 40$ fs. These plots and also the comparison with Fig. 4(d) of the main text where we used or $T_2 = 20$ fs, demonstrates that the choice of the dephasing time T_2 has no significant influence on the main results. A longer dephasing time T_2 just leads to a smaller linewidth which thus allows to resolve some additional structures, in particular, for weak field strengths. Those are, however, neither visible in experiment nor relevant for the findings described in the main text.

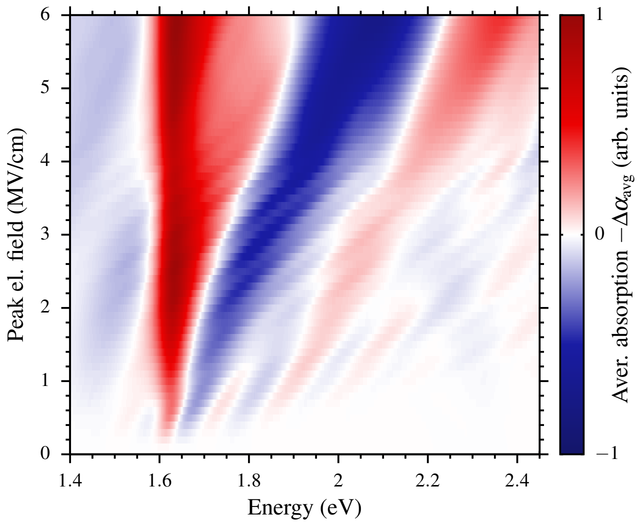

Supplementary Figure 9 1D absorption change, $-\Delta\alpha_{\mathrm{avg}}$, averaged for a pure cosine model band structure (without the function g, see methods, which was introduced to fit the effective mass) from $\overline{\Gamma \mathrm{Z}}$ to $\overline{\Gamma \mathrm{A}}$ direction for a THz pulse centered at $t = 0$ and various field strengths. In comparison to the band structure which includes the effective mass, the averaged transition energy for the $\overline{\Gamma \mathrm{Z}}$ direction is slightly increased from 1.954 eV to 1.975 eV and therefore the main transition to the central Wannier-Stark state starts at a slightly higher energy. Clearly, comparing to Fig. 4(d) of the main text demonstrates that the overall characteristics remain unchanged and thus do not depend on the exact modeling of the band structure.

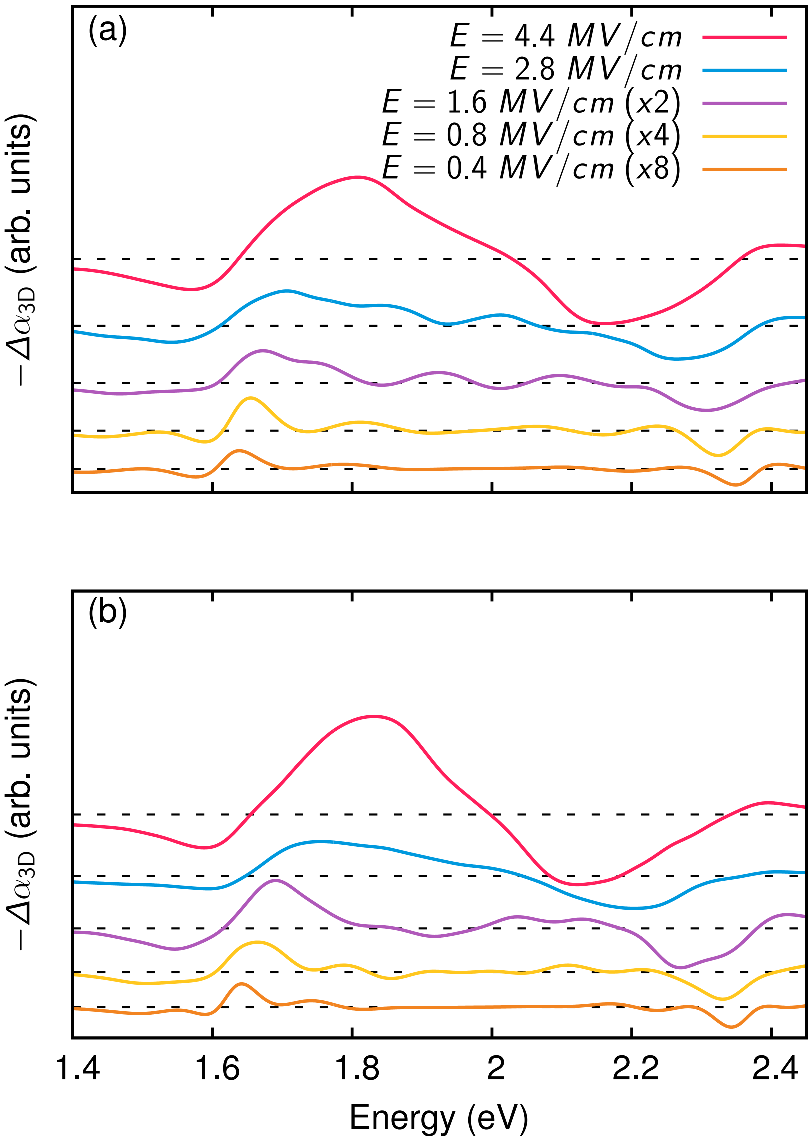

Supplementary Figure 10 Negative change of the optical interband absorption $-\Delta\alpha_{\overline{\Gamma Z}}$ for the excitation with a THz pulse with various peak field strengths, from a cosine band modeling along $\overline{\Gamma Z}$ direction. The pulse duration $\bar{T}$ is 150 fs and the dephasing time is $T_2 = 20$ fs. As attempts to reproduce the THz oscillations observed in the experiments (Fig 2a, Fig 4b), we modify the dipole moment matrix element, μ, to be stronger at lower band edge. The model dipole moment matrix element here is, $\mu \propto 1.62\ \mathrm{eV}/E_{cv}(k)$. (a) Calculation for a THz frequency of 30 THz. (b) Calculation for a THz frequency of 20 THz.

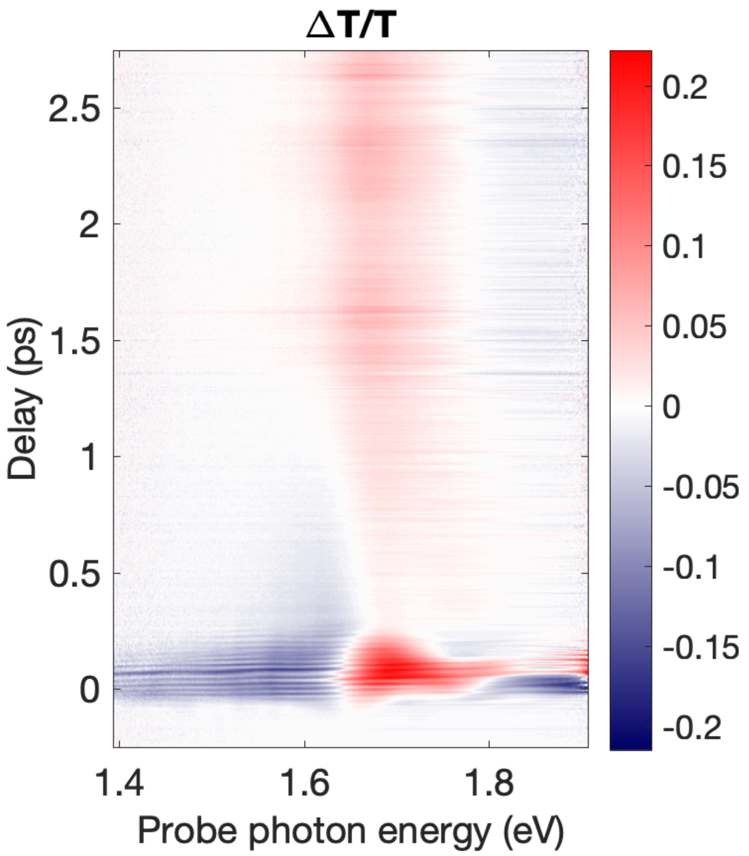

Supplementary Figure 11 Contributions from free carriers generated via interband tunneling after biasing with a 6 MV/cm THz field. Differential transmission changes, $\Delta T/T$, against probe photon energy and over an extended time delays between pump and probe pulses up to 3 ps. For times after the THz pulse the buildup of bleaching above the band gap is visible, however, these transmission changes are well separated from and smaller than the ones measured during the excitation.

Supplementary References

1. Kim, H. *et al.* Direct observation of mode-specific phonon-band gap coupling in methylammonium lead halide perovskites. *Nat. Commun.* **8**, 687 (2017).
2. Karakus, M. *et al.* Phonon-Electron Scattering Limits Free Charge Mobility in Methylammonium Lead Iodide Perovskites. *J. Phys. Chem. Lett.* **6**, 4991–4996 (2015).
3. Schmidt, C., Bühler, J., Heinrich, AC. *et al.* Signatures of transient Wannier-Stark localization in bulk gallium arsenide. *Nat Commun* **9,** 2890 (2018).
4. Egri, I. A simple model for the unified treatment of Wannier and Frenkel excitons. *J. Phys. C: Solid State Physics* **12**, 1843 (1979)
5. Meier, T., von Plessen, G., Thomas, P. & Koch, S. W. Coherent Electric-Field Effects in Semiconductors. *Phys. Rev. Lett.* **73**, 902 (1994)

6. Umari, P., Mosconi, E. & De Angelis, F. Relativistic GW calculations on $CH_3NH_3PbI_3$ and $CH_3NH_3SnI_3$ Perovskites for Solar Cell Applications. *Sci Rep* **4,** 4467 (2014).
7. Haug, H. & Koch, S. W. Quantum Theory of the Optical and Electronic Properties of Semiconductors. (WORLD SCIENTIFIC, 2009).
8. Yang, Y., Yang, M., Zhu, K. *et al.* Large polarization-dependent exciton optical Stark effect in lead iodide perovskites. *Nat Commun* **7,** 12613 (2016).
